# Supplementary material for: Empirical methods for controlling false positives and estimating confidence in ChIP-Seq peaks
Source: BMC Bioinformatics. 2008 Dec 5;9:523. doi: 10.1186/1471-2105-9-523 (PMC2628906; doi:10.1186/1471-2105-9-523)
Supplement: Additional File 1 — A variety of html documents from the USeq web site detailing the available applications, their best usage, output file type descriptions, command line menus, etc. [file 1471-2105-9-523-S1.zip › USeqUserGuides/JavaDoc.zip!/JavaDoc/trans/tpmap/package-frame.html]

trans.tpmap


trans.tpmap

Classes 
  
BlastFilterResult
  
CollapseTPMap
  
ConvertTPMapsToFeatures
  
FilterTPMapByRegions
  
IntensityFeature
  
MapFeature
  
MapSplitter
  
MummerMapper
  
OligoFlipper
  
Stat1lq
  
StatTPMap
  
StatTPMapControlSeqs
  
TPMapDensityReducer
  
TPMapDuplicateFilter
  
TPMapFeature
  
TPMapLine
  
TPMapOligoBlastFilter
  
TPMapProcessor
  
TPMapSort
  
TPMapStripHighLowGC
  
WindowMaker
